# Polyaryletherketone Based Blends: A Review

**DOI:** 10.3390/polym15193943

**Published:** 2023-09-29

**Authors:** Adrian Korycki, Fabrice Carassus, Olivier Tramis, Christian Garnier, Toufik Djilali, France Chabert

**Affiliations:** 1 LGP-ENIT-INPT, Université de Toulouse, 47 Avenue d’Azereix, 65016 Tarbes, France; adrian.korycki@enit.fr (A.K.); fabrice.carassus@groupe-lauak.com (F.C.); olivier.tramis@outlook.com (O.T.); christian.garnier@enit.fr (C.G.); 2 LAUAK Service Innovation, 8 Rue Louis Caddau, 65000 Tarbes, France; toufik.djilali@groupe-lauak.com

**Keywords:** polymer blend, miscibility, thermal transition, crystallization, mechanical properties

## Abstract

This review aims to report the status of the research on polyaryletherketone-based thermoplastic blends (PAEK). PAEK are high-performance copolymers able to replace metals in many applications including those related to the environmental and energy transition. PAEK lead to the extension of high-performance multifunctional materials to target embedded electronics, robotics, aerospace, medical devices and prostheses. Blending PAEK with other thermostable thermoplastic polymers is a viable option to obtain materials with new affordable properties. First, this study investigates the miscibility of each couple. Due to different types of interactions, PAEK-based thermoplastic blends go from fully miscible (with some polyetherimides) to immiscible (with polytetrafluoroethylene). Depending on the ether-to-ketone ratio of PAEK as well as the nature of the second component, a large range of crystalline structures and blend morphologies are reported. The PAEK-based thermoplastic blends are elaborated by melt-mixing or solution blending. Then, the effect of the composition and blending preparation on the mechanical properties are investigated. PAEK-based thermoplastic blends give rise to the possibility of tuning their properties to design novel materials. However, we demonstrate hereby that significant research effort is needed to overcome the lack of knowledge on the structure/morphology/property relationships for those types of high-performance thermoplastic blends.

## 1. Introduction

High-performance thermoplastic (HPT) polymers lead the extension of advanced tailored applications such as embedded electronics, robotics, aerospace, medical devices and prostheses. In addition, HPT research and growth are driven by their use in structural composite materials, in which thermoplastics are an option for the matrix, offering significant weight savings and time-to-market reduction compared to thermoset composites or incumbent steel. Other widespread applications of HPT are as membranes from the barrier, filtration, osmosis and as templates for chemical reactions, such as proton exchange membrane fuel cells.

Thermostable polymers are defined as materials with heat resistance in continuous uses at 200 °C or above and resistance to aging in a thermo-oxidative environment. Only a few thermoplastics reach these outstanding properties, their common feature being the presence of aromatic groups in their chemical backbone. Among them, polyaryletherketones have been developed since the 1980s and have demonstrated some of the longest lifespans when submitted to thermo-oxidative aging [1]. PAEK are semicrystalline polymers displaying a large range of melting temperatures (T_m_) from 320 °C for polyetheretheretherketone (PEEEK) to 390 °C for polyetherketoneketone (PEKK), with their crystalline morphology, the kinetics of crystallization and properties. The most popular is polyetheretherketone (PEEK). PEEK has a glass transition temperature (T_g_) of 143 °C and a melting temperature of 335 °C. Its maximum operating temperature is from 250 °C to 260 °C and its processing temperature is from 370 °C to 400 °C [2]. The maximum achievable crystallinity is 48% [3]. Due to the fast kinetics of crystallization, it was reported that the amorphous state of pure PEEK can be obtained only when cooled at very high cooling rates, nearly 1000 K·min^−1^ [4].

All PAEKs offer a compromise between thermal stability, mechanical properties, chemical resistance and durability. The enhancement of these features can lead to the development of cutting-edge applications [5]. Ideally, one material should fit all the targeted properties, namely “multi-functional” material. Blending two polymers may be the easiest option to design such new materials. Indeed, two or more thermoplastics, often chosen such as their properties complement one another, yield a new material with synergetic properties.

When blending thermoplastics, the miscibility of the two polymers is often required, especially in load-carrying applications, since no miscibility gives a weak interfacial adhesion, resulting in a poor stress transfer from one phase to the other, leading to early fracture. In some specific cases, blends of immiscible polymers may be used, such as in chemical environments or tribological applications. The first polymer has poor chemical resistance or wear but it serves as the mechanical structure and the added polymer acts as a barrier or coating, giving a blend with improved targeted properties [6].

Blending PAEK to another HPT is a possible way to obtain materials with new affordable properties. However, the incorporation of an amorphous polymer in a semicrystalline matrix could induce an increase in the amorphous phase, thus reducing the amount of the crystalline phase. In most cases, the amorphous phase is considered as a diluent for the crystalline phase. Thus, the more amorphous content, the more difficult the crystallization of the crystalline phase. It is then possible to affect the crystallization kinetics by blending an amorphous polymer with a semicrystalline one.

This review focuses on blends of polyaryletherketone-based thermoplastics with various high-performance thermoplastic polymers. Despite their uses and rising interest, no review article has addressed the topic of high-performance thermoplastics blends until now. This work reviews research works started over 30 years ago. It is important to point out that some of these studies are former. Scientific techniques and knowledge could have evolved since then. We have carefully examined some hypotheses put forward in these papers. More specifically, the main topics of this review will be the miscibility of PAEK with HPT, their effect on PAEK crystallization, thermal transitions (glass transition and melting) and the mechanical properties of the blends.

## 2. Presentation of Blends Components

In this section, the polymer blends are classified according to their miscibility and then the chemical structures and properties of PAEKs and other high-performance thermoplastics are briefly presented.

### 2.1. Classes of Blends

Many attempts have been constructed to classify the polymer blends: miscible, immiscible, compatible, incompatible, partially miscible, etc. Our goal is not to review terminology or to discuss which definition is best suited to each situation. We rather define the framework of this review in comprehensive terms, for which we will use the following terms to classify the PAEK/HPT blends: miscible, partially miscible and immiscible. Miscible blends behave in a single phase and exhibit a single glass transition temperature. Partially miscible blends exhibit miscibility to an extent, as a function of blend composition or process parameters. It is worth mentioning that, whatever the polymer added to PAEK, the miscibility depends on the molecular mass. However, this information is barely provided in the reviewed articles. Finally, the blends that do not fall into the previous categories are classified as immiscible blends. Table 1 shows the common PAEK/HPT blends found in the literature. It is noteworthy that PEI is the only HPT that forms a fully miscible blend with various PAEK in the melted state. However, PEI comes in different conformations, and some of them are not miscible with PAEK. This point will be developed in Section 6.

### 2.2. Presentation of PAEK

**Figure 1:**
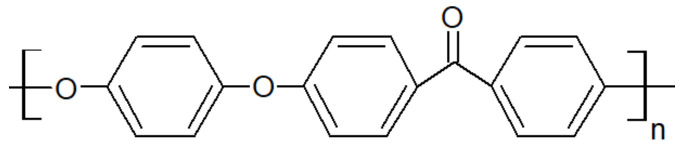
Structural formula of PEEK.

**Figure 2:**
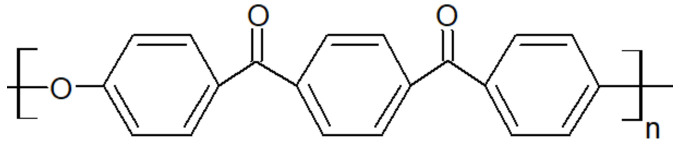
Chemical structure of PEKK.

**Figure 3:**
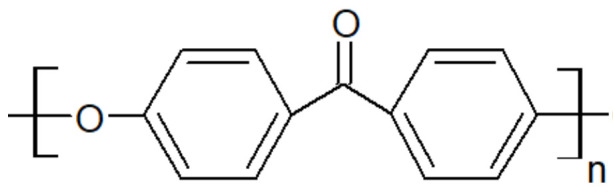
Scheme of PEK unit.

Polyaryletherketones are semicrystalline high-performance thermoplastics with strong molecular rigidity of their repeating units. They demonstrate high-temperature stability, chemical resistance and high mechanical strength over a wide temperature range. PEEK (Figure 1), PEKK (Figure 2), PEK (Figure 3) and others have similar crystalline structures of two-chain orthorhombic packing [7] but not the same ether/ketone content [8].

Polyetheretherketone combines the strength of 98 MPa and the stiffness of 125 MPa with a very good tensile fatigue of 97 MPa, thermal and chemical resistance (including the majority of organic solvents, oils and acids). Its mechanical properties remain stable up to temperatures of about 240 °C [9].

Polyetherketoneketone is a polymer with high heat resistance above 300 °C, chemical resistance and an ability to withstand high mechanical loads from 88 MPa to 112 MPa [10,11,12]. This polymer is synthesized in various formulations with individually unique properties. The PEKK formulations are expressed by the ratio of the percent of terephthaloyl (T) to isophthaloyl (I) moieties used during the synthesis that created the polymer. The T/I ratio affects the melting point ranging from 305 °C to 360 °C, the glass transition temperature from 160 °C to 165 °C and the crystallization kinetics [13].

Polyetherketone is characterized by very good material properties such as a tensile strength of 110 MPa, an elastic modulus of 4200 MPa and an elongation at break of 35%. It has a high resistance to abrasion and increased compression strength of 180 MPa at higher temperatures [14].

**Figure 4:**
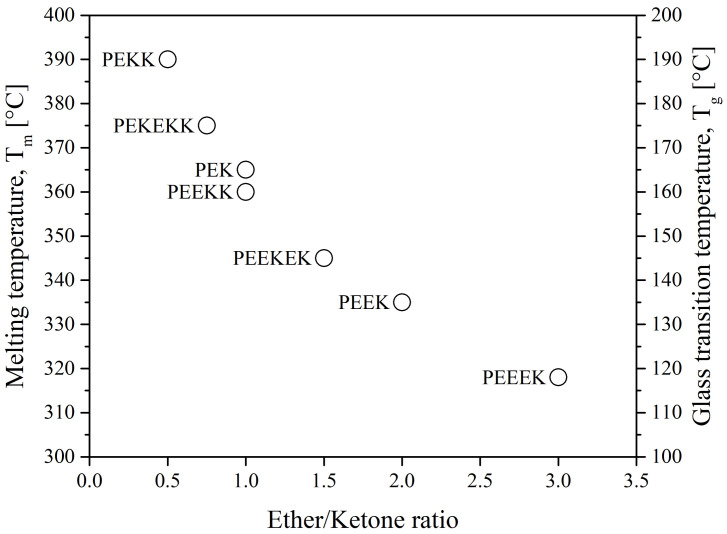
The melting temperature and glass transition temperature of PAEK as a function of ether/ketone ratio [16].

The properties of polyaryletherketones are due to the occurrence of phenylene rings linked via oxygen bridges (ether, R–O–R) and carbonyl groups (ketone, R-CO-R) in different configurations and proportions. The glass transition temperature, T_g_, and the melting temperature, T_m_, of the polymer depend on the ratio and sequence of ethers and ketones. They also affect its heat resistance and processing temperature. The lower the ratio of ether/ketone, the more rigid the polymer chain is and the higher the T_g_ and T_m_ as seen in Figure 4. As a consequence, the processing temperature ranges from 350 °C to 400 °C [15]. The main suppliers are Victrex, Arkema, Solvay, Evonik, Sabic and Gharda.

**Figure 5:**
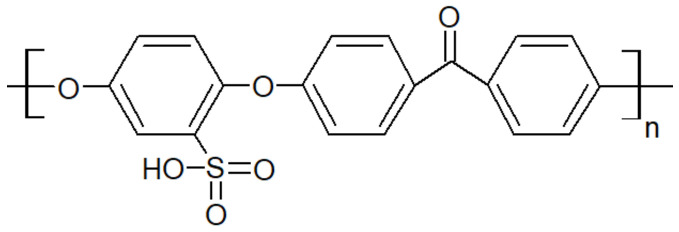
Sulfonated PEEK structure.

Another type of PAEK was launched recently on the market by Victrex, with a lower melting temperature while keeping the same glass transition, maned as LM-PAEK (LM: low melting). The typical trend for T_g_ and T_m_ as seen in Figure 4 is not valid anymore, due to the higher rigidity of its monomers. Furthermore, to increase the hydrophilicity of PEEK materials, charged groups are introduced using sulfuric acid into the polymer chains to make them ion-exchangeable. Sulfonation aids in the transport of cations and increases the hydrophilicity of the polymer. Sulfonated PEEK is presented in Figure 5.

### 2.3. Presentation of HPT Usually Blended with PAEK

**Figure 6:**
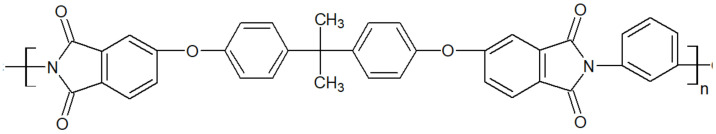
Schematic structure of PEI Ultem 1000.

Polyetherimide is an amorphous polymer whose chemical structure of grade Ultem 1000 from Sabic is seen in Figure 6. It is renowned for being inherently flame retardant. Ultem 1000 resin in an unreinforced general-purpose grade offers a high strength of 105 MPa and a modulus of 3.2 GPa and a broad chemical resistance up to high temperatures of 170 °C while maintaining stable electrical properties over a wide range of frequencies [17,18]. The ketone groups in its backbone render it more flexible than polyimides, hence better processability. Similar to other amorphous thermoplastics, its mechanical strength decreases fast above its T_g_ at 215 °C. PAEK/PEI blends have been reported to be miscible, especially the binary blend PEEK/PEI. The latter is commonly used in PEEK composite parts [19,20,21], where the PEI is a joining agent, a method referred to as “Thermabond” [22]. Also, PEI is used as an energy director, meaning interfacial film, to assemble carbon fiber/PAEK composites through ultrasonic welding [23]. PEEK/PEI blends have also found application as tribological material [24], biomedical implants [25] and lightweight foam structures [26]. Since this blend has been reported to be miscible at all compositions in the amorphous state, it has attracted considerable attention to further the fundamental understanding of miscible blends [27,28,29,30,31].

**Figure 7:**
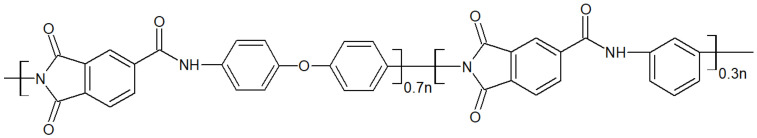
Chemical structure of the PAI Torlon^®^ 4000T.

Most studies with polyamideimides mention Torlon^®^ 4000T from Solvay, whose chemical structure is presented in Figure 7, as well as other PAI Torlon^®^. Torlon^®^ 4000T is the unfilled PAI powder mainly for adhesive applications [32]. Some authors have synthesized their PAI [33] with specific properties. Their mechanical, thermal and oxidative properties make them suitable for various applications thanks to a high glass transition temperature of around 275 °C [34].

**Figure 8:**
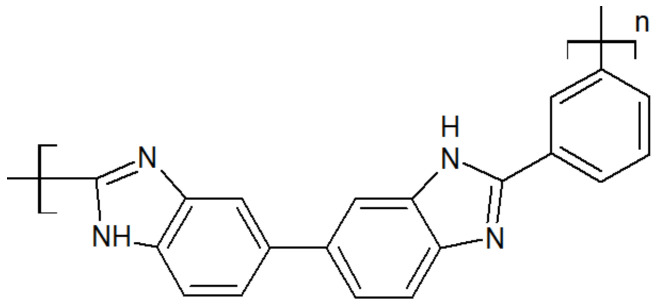
Scheme of a PBI unit.

The chemical structure of polybenzimidazole is presented in Figure 8. Its rigid structure gives excellent thermal and mechanical properties, such as a melting point above 600 °C [35] and a glass transition of 435 °C [36]. It has the highest tensile strength among high-performance polymers, up to 145 MPa, and offers good chemical resistance [35,37,38,39]. However, it has a large water uptake of 15 wt.%, and the imidazole ring in the repeat unit may be subjected to hydrolysis, reducing its lifetime in applications such as fuel cells [40].

**Figure 9:**
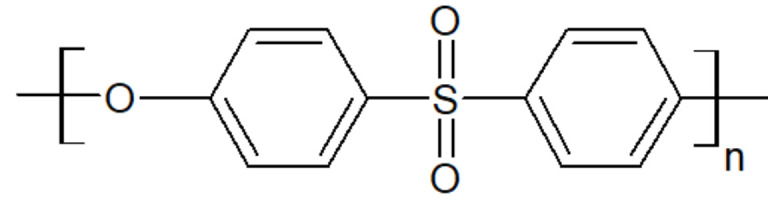
The repeat unit of a PES.

Polyethersulfone, whose chemical structure is sketched in Figure 9, is an amorphous thermoplastic with a T_g_ between 190 °C and 230 °C, yielding high thermal stability. It also offers high mechanical rigidity and creep resistance, which make it a good candidate to blend with PAEK [41].

**Figure 10:**
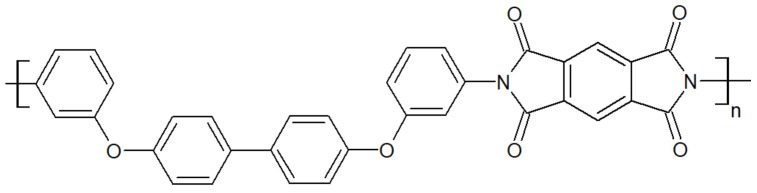
TPI repeat unit.

Thermoplastic polyimides, whose structure is depicted in Figure 10, display high mechanical properties of 128.7 MPa at tensile strength and 14.2% at elongation at break for TPI-4 [42]. They have one of the highest continuous operating temperatures for an unfiled thermoplastic of 240 °C [43]. Their T_g_ is at 250 °C. The main drawbacks of TPI are their high melt viscosity and low chemical resistance, making their processing challenging. Modification of the aromatic backbone by the inclusion of flexible functional bonds has enabled the synthesis of polyimide with a lower melt viscosity [44,45]. While TPIs are amorphous (Matrimid 5218, LaRC-TPI, Extem XH/UH), semicrystalline TPIs (with a crystallinity at 20%) were developed as an alternative, such as the so-called “New-TPI” by Mitsui Toatsu Chemical, Inc. (Tokyo, Japan) [46], later renamed “Aurum” or “Regulus” [47] depending on its end-use. Both amorphous and semicrystalline TPI suffer from low processability, even though the N-TPI has better overall mechanical properties and chemical resistance than TPI. Blending TPI or N-TPI with PAEK is an interesting way to improve the processability of the former, despite their immiscibility with PAEK.

**Figure 11:**
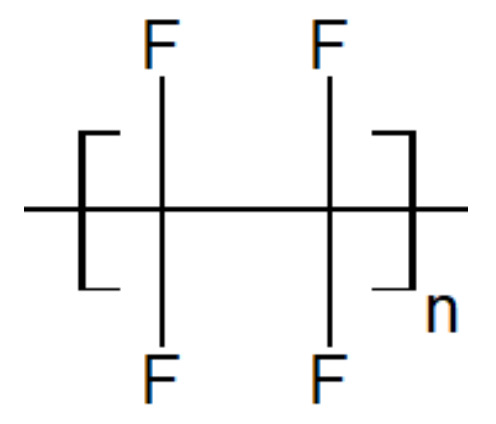
Schematic structure of the PTFE unit.

Polytetrafluoroethylene is a synthetic fluorocarbon with a high molecular weight compound consisting of carbon and fluorine. Their structure is shown in Figure 11. It is highly hydrophobic, biocompatible and widely used as a solid lubricant thanks to its low friction coefficient. Concerning engineering applications, PEEK is wear-resistant but may suffer from wear loss or high friction coefficient at elevated temperatures. Blending PEEK with PTFE is a way to improve the tribological performances of PAEK-based blends. But PTFE is extremely viscous in the melted state, which complicates its processability. Its glass transition temperature is around 114 °C and its melting point is 320 °C and it starts deteriorating above 260 °C, making it difficult to blend with PAEK.

**Figure 12:**
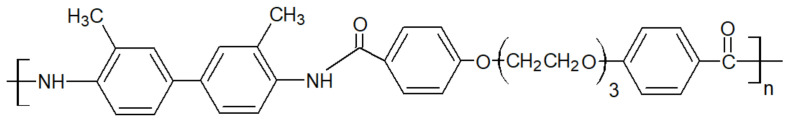
Repeat unit of TLCP.

Liquid crystal polymers are characterized by the properties of liquid crystal, which can be those of conventional liquids or those of solid crystals. For example, a liquid crystal may flow like a liquid, but its molecules may be oriented in a crystal-like way. Those thermoplastics showed up on the market in the 1980s. Thermotropic LCP, shown in Figure 12, is melt processable, while lyotropic LCP with a higher chain rigidity and intermolecular bonding is spun from a solution such as sulfuric acid. During melt processing, the rigid molecules with units disrupt chain linearity and pack slightly to reduce the melting point. It has been reported that the blends with LCP often tend to be immiscible but have a range of useful properties. TLCP have been blended with many thermoplastics including PEEK [48].

## 3. Thermodynamics of Miscibility and Morphologies

Polymer–polymer theory of miscibility is based on the Flory–Huggins theory for a solution, for which, ideally, each part of the solute fills a space in the solution that it is soluble in, e.g., the so-called “lattice model” [49]. Polymers, and especially HPT, are at best semirigid due to their aromatic backbone. The Flory–Huggins theory still serves to describe the polymer–polymer mixture. The free energy of mixing depends on combinatorial entropy since the polymer of the mixture cannot fill all the space offered by the lattice due to its restricted degree of freedom. This effect is taken into account by considering the volume of the polymer, i.e., steric effects [50]. The free energy of mixing depends also on the interaction between like and unlike pairs of monomers, which is an enthalpic contribution. Usually, since macromolecules contain a large number of monomers, the repulsion between macromolecules is large [51]. The strength of this interaction, as the difference between like and unlike pairs, is defined as the interaction Flory–Huggins parameter, χ. The free energy of mixing is usually positive, i.e., unfavorable to mixing. However, strong specific interactions (dipole–dipole, ion–dipole, hydrogen bonding, acid-base or charge transfer) [52] may occur, and these will counterbalance the unfavorable entropic (steric) and enthalpic (van der Walls) interactions to give miscibility. Additionally, the free energy of mixing must have a positive curvature (in other words, increased differential growth at the center, for example, the surface of the sphere) at any concentration to give a miscible blend concerning the negative enthalpic term. Usually, this is accomplished by specific interactions, such as dipole–dipole and hydrogen bonding [53]. Such interactions are highly dependent on temperature; as a consequence, the miscibility depends on the temperature. This dependency is evidenced by miscibility diagrams depicting an upper critical solution temperature (UCST) or a lower critical solution temperature (LCST).

Partially miscible blends have a negative curvature (in other words, increased differential growth at the edges, for example, “saddle”) for some concentrations on a miscibility diagram [54]. For example, PEEK/PES blends are partially miscible; their miscibility depends on the processing method. A probable cause, in view of the discussion above, would be that their respective conformation does not allow the macromolecules to interact attractively. However, sulfonated PEEK is fully miscible with PES, mainly due to the presence of sulfonate groups, which would specifically interact with the likes on the PES backbone. Another example would be PEEK/PBI blends, where steric repulsion would be the main reason for their immiscibility. Nevertheless, SPEEK is fully miscible with PBI, mainly due to the acid-base interaction between the basic, amide groups of the PBI and the acid, sulfonate groups of the SPEEK.

When functionalization is not possible, immiscible polymers may be compatibilized. The addition of a compatibilizer is the main route to obtain melt-mixed polymer blends [55,56]. The compatibilizers may be classified into two categories:

- Nanoparticles, which are partially or fully miscible with the polymers in the blend [57]. Usually, they are amphiphilic, “Janus” nanoparticles [58] aimed at the interface to reinforce it and promote the stress transfer between the two phases;
- Block copolymers: For instance, PET/PP blends are often compatibilized by PP-*b*-MA (MA: maleic anhydride) copolymers, where the *block* MA is miscible with PET. Gao et al. [59] compatibilized PEEK/PI blends with PEEK-*b*-PI block copolymers. In any case, the length of the blocks and the number of copolymers control the stability and the final morphology of the blends. It is worth noting that the choice of copolymers available to compatibilize PAEK with another HPT is very narrow.

**Figure 13:**
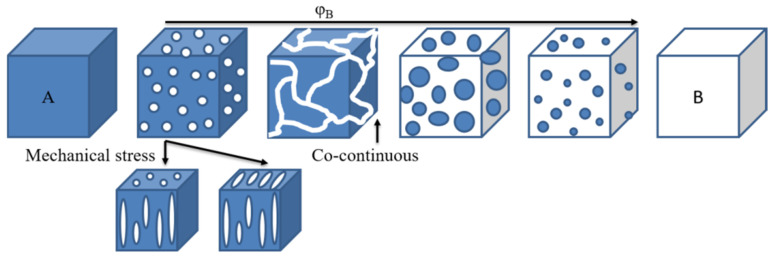
Basic types of phase structures in polymer blends.

Immiscible blends exhibit a two-phase morphology, where each phase is a rich phase of one component, whereas miscible blends should behave as a single-phase material. Compatibilization leads to fine dispersion of one of the phases into the other, depending on the composition. Partially miscible blends have a spectrum of morphologies depending on the composition and processing parameters, as in Figure 13. Of interest is obtaining a co-continuous structure [60]. Indeed, even if the blend has an immiscible state, a co-continuous morphology may give a blend with mechanical properties better than expected. Thus, if blending two polymers leads to an immiscible blend, but they have complementary properties (for instance, thermal stability or solvent resistance), one may want to process them with a set that gives a co-continuous morphology to obtain enhanced properties. Such morphologies are expected when blending PAEK with other thermoplastics, likewise with conventional polymer blends.

**Figure 14:**
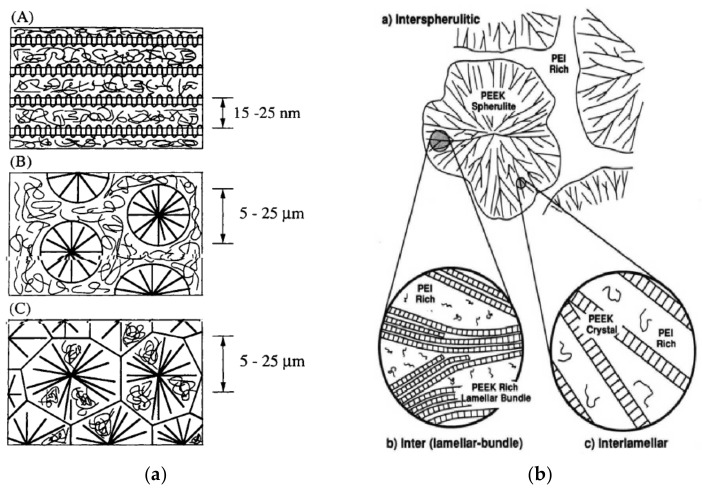
Schemes of the morphologies in the semicrystalline/amorphous blends. On the left (**a**), the spherolite sizes, with permission from Wiley [29] and right (**b**), macromolecular organization inside a spherulite, with permission from ACS Publications [28].

Moreover, due to the crystallization of PAEK, the resulting morphology depends on the crystallinity generated in the blend. In the PAEK/HPT blends, the HPT are mostly amorphous, so the semicrystalline/amorphous blends may develop three main configurations [28,29,61,62] as shown in Figure 14:

- Interlamellar, where the semicrystalline polymer forms pure lamellas, alternating with an amorphous-rich phase (Figure 14A, left or Figure 14b, right);
- (Inter)spherulitic, where the spherulites develop in an amorphous-rich matrix (Figure 14B, left or Figure 14a, right);
- Interfibrillar or inter(lamellar-bundle), where the amorphous polymer may be trapped between bundles of the semicrystalline polymer (Figure 14C, left or Figure 14b, right).

The competition between the diffusion of the amorphous component out of the crystal domains and the crystal growth is the controlling factor, while strong segmental interactions may hinder such competition. In the case of weak interactions, interlamellar morphologies (cases Figure 14A and Figure 14C) may be favored if the amorphous component diffuses fast and the crystal growth is slow. Conversely, trapping of the amorphous component may occur, leading to spherulitic morphologies.

## 4. Evaluation of the Miscibility

Citing Olabis et al. [52], “The most commonly used method for establishing miscibility in polymer-polymer blends or partial phase mixing in such blends is through the determination of the glass transition (or transitions) in the blend versus those of the each separated constituent. A miscible polymer blend will exhibit a single glass transition between the T_g_’s of the components with a sharpness of the transition similar to that of the components. In cases of the borderline, miscibility broadening of the transitions will occur”, supported by a citation from MacKnight et al. [63], “Perhaps the most unambiguous criterion of polymer compatibility is the detection of a single glass transition whose temperature is intermediate between those corresponding to the two-component polymers”. Thus, the miscibility of polymer blends is most often checked by the measurement of the T_g_ of the blends. The blends of amorphous and semicrystalline polymers have a broader T_g_ than the pure components, attributed to a wider distribution of the amorphous domains. From thermodynamical considerations, such as those developed by Couchman [64], T_g_ was found to depend on the composition of the blend and usually increases with it. Several equations have been developed on those assumptions to describe the T_g_ dependency over the composition. The well-known Fox equation [65] (Equation (1), where w_i_ is the weight fraction) may be the most widely used for polymeric blends,

1/T_g,mix_ = Σ_i_(w_i_/T_g,i_) (1)

whereas more sophisticated equations, such as the Gordon–Taylor equation [66], have been developed based on the same theoretical basis, but with a different hypothesis where the equation (Equation (2)) is reduced by Wood [67] for a two-component system. Utracki and Jukes proposed an equation that follows T_g_ versus weight fraction dependencies for miscible blends and plasticized systems [68]. For partially miscible blends, they introduced an empirical parameter, K, describing the deviation from the assumed negligibility of entropy of mixing.

T_g,mix_ = (w_1_T_g1_ + Kw_2_T_g2_)/(w_1_ + Kw_2_) (2)

More equations derived from those considerations may be found in the report of Aubin and Prud’homme [69].

The miscibility of the blends is often assessed by differential scanning calorimetry (DSC). This technique is quite accurate and it requires a small piece of samples. The blends are considered miscible if only one T_g_ appears on the DSC thermogram. In other cases, the immiscibility is proved by the presence of unchanged T_g_ of the pure components, in partially miscible blends, two T_g_ dependent on the composition appear [70]. Dynamic mechanical analysis (DMTA) may also be used to assess glass transitions (referred to as T_α_) at a macroscopic scale. The shape at the T_α_ point in the loss factor (E″) signal may be an indicator of miscibility. In this case, a sharp T_α_ will be observed. A broader peak of the T_α_ would indicate phase separation to an extent, as the broad transition would encompass two distinct amorphous phases. If the two components display T_α_ that are close to each other, its measurement would yield a single T_α_ even if the polymers are not miscible. In this case, the sharpness of the E″ peak signal may help to distinguish miscible from immiscible blends. Another method of assessing miscibility would be the measure of the lower critical solution temperature (LCST). Its simplest measurement would be the detection of the cloud point, where phase separation would produce opacity in an otherwise transparent blend in the melt state. This method, however, may not be applicable for semicrystalline blends if crystallization occurs before crossing the LCST, i.e., for blends with a high percentage of a semicrystalline component.

Citing Wang and Cooper [71], “It is well known that compatible polymer blends are rare”. Due to a lack of thermodynamic compatibility, only a few polymer blends are intimately miscible, i.e., miscible at a segmental level [72].

## 5. Elaboration of PAEK Blends

Two methods exist to obtain polymer blends: mixing the components of the blend in the melt state or by dissolution. In the latter case, both components are dissolved in a common solvent. PAEK, however, may suffer chemical modification if dissolved in strong acids, a mainly undesired functionalization. Therefore, controlled functionalization of PAEK is performed before dissolution. Both methods are explained in the following.

### 5.1. Melt-Mixing

Components of the blends are mechanically processed in the melted state, either by conventional mixing using an internal mixer or double-screw extrusion. Further, the so-obtained blends are processed through compression molding or injection molding to obtain specimens or final parts. Melt-mixing enables the independent control of various experimental parameters, such as melt temperature, pressure, residence time, shear rate and cooling rate. This method is appropriated for the industry as an environmentally friendly process because of the absence of the solvent. Additionally, melt-mixing may produce a finely dispersed phase [73] or co-continuous structure [74] depending on the parameters chosen. However, in the case of HPT blends, the residence time and melt temperature have to be carefully selected to prevent the degradation of one or both components upon processing.

During the polymer processing of blends, the viscosity mixing rules should be considered [75]. Some equations give the viscosity of the blend as a function of component viscosities, blend composition and shear rate. The flowing ability is associated with the viscosity of the molten polymers. The viscosity is highly influenced by the temperature and shear rate of molten polymers [76]. Molten polymers generally exhibit shear-thinning behavior with a Newtonian plateau at a low shear rate, standard polymer processing such as injection or extrusion involves shear rates above 1000 s^−1^. Bakrani Balani et al. [77] reported a decrease in the viscosity from 11,000 Pa·s to 1200 Pa·s for PEEK at 0.03 rad·s^−1^ and 100 rad·s^−1^, respectively, at 350 °C. Moreover, the viscosity of thermoplastics is highly influenced by the temperature: Bakrani Balani et al. [78] also reported a decrease in the viscosity from 11,000 Pa·s to 5300 Pa·s for PEEK from 350 °C to 400 °C.

The rheological behavior of PAEK and their blends have been barely published [79,80,81]. Molten PEEK demonstrates a classical behavior similar to conventional thermoplastics. The behavior of blends is more complicated; only a few articles analyze their rheological properties [19,82,83,84,85]. The blends do not reach a Newtonian plateau in the experimental frequency range, continuously increasing with decreasing frequency. This may be due to the morphological organization of immiscible domains [86]. Like any copolymer, it self-organizes into spheres, cylinders, lamellae or network-like morphology, depending on the relative polymer-block chain lengths, Figure 13. Such morphologies strongly impede the flow behavior and relaxation characteristics of the material.

Processing PAEK through melt-mixing requires knowledge and practice. Indeed, molten PAEK is subjected to thermo-oxidative degradation, resulting in viscosity increases for the highest residence times. As an example, the viscosity of PEEK slowly increases from 1600 Pa·s to 1750 Pa·s at 330 °C; at the higher temperature of 390 °C, the viscosity increases from 750 Pa·s to 900 Pa·s. It was observed that at 370 °C, the PEEK modulus increases after less than 4 min if under air atmosphere [87].

For these reasons, understanding the thermal decomposition mechanisms of aromatic polyketone, consisting of ketone and aromatic moieties, is essential. The kinetic parameters of the mechanisms involved during decompositions were first studied by Day et al. [88,89]. An isothermal weight-loss method in air and nitrogen atmosphere was investigated and the degradation was observed between 575 °C and 580 °C. Char yields of this polymer are above 40% [90]. The thermal degradation of PEEK occurs in a two-step decomposition. The first step is a random chain scission of the ether and ketone bonds [91]. Carbonyl bonds create more stable radical intermediates, which would be expected to predominate. The second step is due to the oxidation of the carbonaceous char formed. Oxidation of pure PEEK occurs at around 700 °C.

### 5.2. Solution Blending—Sulfonation of PAEK

One of the main advantages of PEEK, the chemical resistance, becomes one of its drawbacks when it comes to being dissolved. Indeed, its poor solubility is due to crystallinity and side reactions such as interpolymer crosslinking and degradation [92,93]. One of the methods to increase the solubility of PEEK is to modify its chemical structure to reduce its intrinsic crystallinity. PEEK is only soluble in strong solvents at room temperature and in other solvents (e.g., diphenyl sulfone) at temperatures approaching the melting temperature at 330 (°C). Chemical modifications (e.g., sulfonation) and protonation are the main causes of the solubility of PEEK in strong acids. Sulfonation is a method of choice since it reduces the degree of crystallinity of the PEEK, thus enhancing its solubility. Sulfonation substitutes some atoms of the phenyl rings with sulfonated groups and the position of the substituted carbon depends on the acid used [94]. In the case of sulfuric acid, Shibuya et al. [95] noted that sulfonation only takes place on the phenyl ring sandwiched by two ether groups. The packing and conformation of the backbone are thus modified by the introduction of sulfonate groups as the main cause of the loss of crystallinity. However, the T_g_ of the sulfonated PEEK is increased compared to the neat PEEK. Arigonda et al. [96] reported an increase in the T_g_ from 143 °C for PEEK to 216 °C after sulfonation. This is due to an ionomeric effect, where a polar ionic site can increase intermolecular association [97]. Since sulfonated PEEK is soluble in strong acids (dimethylacetamide, methanesulfonic, dimethylformamide) [97,98], it may be blended with various polymers, such as polyethersulfone, poly(4-vinylpyridine) or polybenzimidazole, as reported by Kerres et al. [99]. These miscible blends are prepared by the dissolution of both components in a common solvent.

However, the same blends prepared via melt-mixing are immiscible. This difference may be explained by other interactions since the backbone of PEEK is different from SPEEK, meaning different miscibility for a given polymer.

## 6. PAEK/PEI Blends Obtained by Melt-Mixing

This section is dedicated to the PAEK/PEI blends. Among all the blends reviewed, they are the only ones falling in the category “miscible in the melt state” and the wider number of articles available in the literature.

### 6.1. PAEK/PEI Blends in the Amorphous State

**Figure 15:**
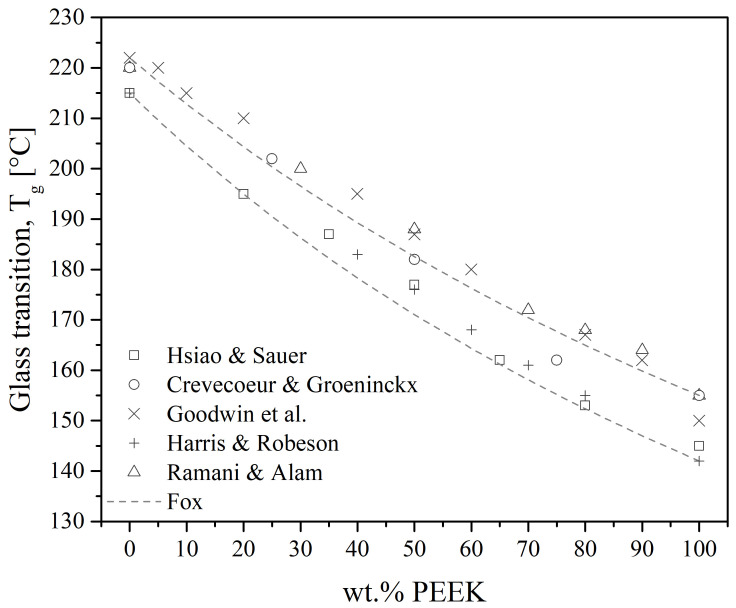
Glass transition temperature as a function of PEEK content in PEEK/PEI amorphous blends. Points represent experimental data from the literature [20,27,29,62,100] and solid lines represent the Fox trend using Equation (1).

Polyetheretherketone/polyetherimide blends are the most studied among the PAEK/PEI blends since they are easily obtained by melt-mixing. They give fully miscible blends at all compositions in the amorphous state. Indeed, all reports reviewed measured a single T_g_ on quenched samples, either using DSC or DMTA [19,20,25,27,28,29,30,61,62,100,101,102,103,104], over the whole range of composition. Good agreement between the values of the measured T_g_ and Fox equation is found in those works, as illustrated in Figure 15.

When having a deeper look, PEI displays several conformations with a great influence on the miscibility with PAEK.

Nemoto et al. [31] showed that the conformation of the phenylenediamines in the PEI backbone greatly influences its miscibility with PEEK. They reported that PEI containing diamines in the meta conformation (i.e., the so-called “Ultem 1000” (*m*-PEI)) were fully miscible, while PEI containing para diamines were immiscible. According to Dingeman et al. [105], the inclusion of meta-linked aryl ether spacer results in a large increase in free volume compared to para linkages. Para-PEI (*p*-PEI) would have a linear macromolecular chain structure with all the junctions between repeat units aligned as if on a straight line. As a consequence, the only degree of freedom would be a rotation around this axis. Oppositely, *m*-PEI would offer a more hooked chain with an overall higher degree of conformation. Thus, the free energy of mixing would be lower for PEEK/*m*-PEI than PEEK/*p*-PEI to the point of giving miscible blends for the former and immiscible blends for the latter. Additionally, phenyl rings in the PEEK are in the para conformation. Thus, in the PEEK/*p*-PEI blend, like pair (*p*-phenyl/*p*-phenyl) interactions would increase and thus increase the enthalpic contribution, such as favoring PEI–PEI chain interaction over PEEK–PEI. On the other hand, the blend of PEEK/*m*-PEI would have an increase in unlike pair interactions, more favorable to mixing. Kong et al. [106] suggested that charge transfer between PEEK and PEI may be the specific interaction that would change the balance toward (im)miscibility. They reported a difference in miscibility between the two PEI conformations. It shall be noted that the PEI may also exist with ortho linkages [105], but such PEI was not investigated further. In the vast majority of scientific studies, *m*-PEI is the chosen conformation, commercially known as Ultem 1000. Overall, it may be supposed that the specific interactions responsible for its miscibility with PEEK would be the same for other PAEK. Indeed, PEK [27,29,107], PEEKK [108,109] and PEKK [27,29,30,107] have been reported to be fully miscible at all compositions in the amorphous state.

### 6.2. Crystallization of PAEK/PEI Blends

Depending on the thermal conditions, the PAEK crystallizes in different morphologies and gives various spherulite sizes.

(1) When cooling PAEK/PEI blends from the melt, only PAEK partially crystallizes. The most significant effect of PEI on crystallization is that chains near the crystals in the amorphous phase are probably less mobile than those farther away, which increases the T_g_ of the amorphous phase in the blend [20,29,62]. Chen and Porter [61] recorded, 30 years ago, a slight negative variation from linearity when measuring the specific volume of the existence of PEI in the interlamellar zone of PEEK crystals, indicating favorable intermolecular interactions between PEEK and PEI. While amorphous blends agree with Fox law, crystalline blends deviate from it, with T_g_ superior to the predicted value. In the range of all blends, the broadening of T_g_ was observed up to 40 wt.% of PEI [61]. The broadening was attributed to a change in the amorphous phase distribution since, upon crystallization, the PEEK has to diffuse to the crystal domains, depleting the amorphous phase [29]. At the same time, the PEI has to diffuse from the crystallizing PEEK to the amorphous phase, creating an enrichment of the amorphous phase in PEI. Thus, the broadening observed could be due to a broad distribution of amorphous PEI, which may comprise free chains (i.e., in the amorphous phase) and trapped chains (i.e., chains at the crystal-amorphous boundary). As already mentioned, blends of PEEK/PEI are reported to be amorphous upon preparation. However, the semicrystalline state is the most likely to be encountered for practical applications. Partial miscibility was revealed in the semicrystalline state by the presence of two distinct glass transitions between 40 wt.% and 90 wt.% of PEEK in the blend [110].
(2) Cold crystallization of PAEK blends was reported not to occur for PEI content from 75 wt.% [61,101], for which the PEI impacts and hinders the PEEK crystallization. Below this concentration, the PEI does not influence the PEEK crystallinity [20]. A decrease in the crystallization with increasing PEI content was measured [20,21,26,61,62,100,104,111,112], attributed to a rejection of PEI into the amorphous domains of PEEK [20]. The final degree of crystallinity of the PEEK reached in the blends did not change with the PEI content and in some cases slightly increased [20,21,26,62,101,111]. The PEEK crystallized similarly to that of pure PEEK, and the melting temperature was not affected by the PEI content.
(3) Isothermally crystallized PEEK/PEI blends exhibited a double melting behavior [62,111,113], seen by two melting peaks on DSC scans. The first peak corresponds to the melting of secondary PEEK crystals, while the second peak, occurring at a higher temperature, corresponded to primary PEEK crystals [29]. Both T_m_s did not depend on the PEI content [26,27,29,62]. As the PEI content increases, the crystal growth of PEEK decreases [29,61], indicating a disruption of PEEK crystallization by the PEI matrix, which induced different nucleation mechanisms [29,111]. Similar effects were observed in PEKK/PEI blends. As PEKK crystallizes slower than PEEK, the PEI may, even more, reduce the nucleation site density, disrupting the PEKK crystallization [29]. The lamellar thickness in all blends was independent of the PEI content; however, it differs for PEKK/PEI blends with about 85 Å and it is higher for PEEK/PEI blends at 100 Å. Torre and Kenny [101] noted that for 50/50 PEEK/PEI blends, the PEI acted as a diluent for the PEEK crystallization, suggesting that PEEK crystallized as if in neat PEEK, since the PEI, in this case, may be completely expelled from the PEEK crystals. This means that blended PEEK and neat PEEK have a similar crystalline structure. However, they may have different growth rates. In another former study [62] on the isothermal crystallization temperature, it can be noticed that deviations observed with T_g_ of crystallized blends containing up to 50 wt.% of PEI were equal to pure PEI, which truly demonstrates the presence of almost 100% of PEI phase in the blend. Some thermograms are indeed incomplete, and the second peak could be assumed to be present on some broader DMTA signals.

**Figure 16:**
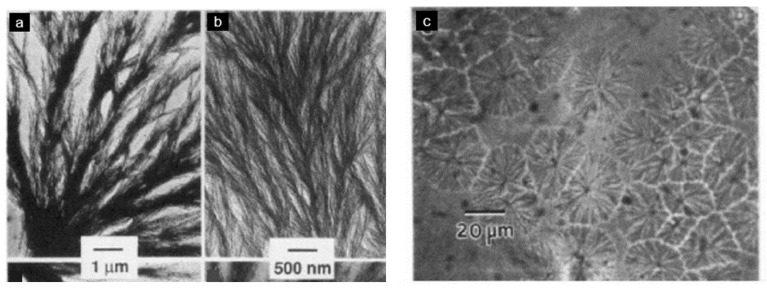
PEI/PEEK morphologies. Left: transmission electron micrographs of inter (lamellar-bundle) segregation, for 25/75 PEI/PEEK blends. Right: optical micrographs of 50/50 PEI/PEEK blends showing interspherulitic morphology, with permission from ACS Publications [28].

Hsiao and Sauer [29] described the general trends for the morphology of PAEK/PEI blends. They supposed that an increase in PEI concentration and a decrease in the degree of undercooling both favor interspherulitic morphology. Good agreement with experimental data was found, as PEEK/PEI blends with a PEI content below 50 wt.% exhibited interlamellar bundle morphologies, revealed by transmission electron microscopy (TEM) by Hudson et al. [28] and by optical microscopy by Hsiao and Sauer [29], as presented in Figure 16. For PEI equal and above 50 wt.%, interspherulitic morphologies appeared, where the PEI was almost entirely excluded from the crystals [29,61,62]. Goodwin et al. [100] described those trends in terms of Avrami analysis. They found that the Avrami exponent shifted downward with an increase in the PEEK concentration, denoting a change from 3D to 2D crystal growth.

The PEEK/PEI blends have been successfully used for the preparation of PEEK hollow fiber membranes [114,115]. Ding and Bikson [114] removed the polyimide phase utilizing a primary amine reagent monoethanolamine (MEA). Porous PEEK membranes, from a 50/50 PEEK/PEI blend, were largely cylindrically shaped and interconnected, quite uniform with a pore diameter of around 10 nm. The degree of crystallinity was determined to be 34% based on the heat of fusion with T_m_ at 325 °C. Huang et al. [115] studied the influence of solvent-induced crystallization during extraction for PEEK/PEI blends with 60 wt.% of PEI. The average crystallite size for membranes before extraction was 5.7 nm with a degree of crystallinity of 39%, while a weak polar solvent dichloromethane and a composite extractant have caused an increase in the crystallite size to 6.2 nm and degree of crystallinity to 46.6%, which shows a strong ability to induce the crystallization of PEEK. A strong polar solvent N-methyl-2-pyrrolidone (NMP) did not change the crystallization parameters. In addition, a composite extractant (80 vol.% NMP, 10 vol.% ethanolamine, 10 vol.% water) had the most dominant distribution of small pores, indicating the strongest extraction ability for interlamellar or interfibrillar PEI. Solvent-induced crystallization can promote further phase separation of PEEK and PEI to form larger pores of membranes.

The degree of crystallinity of PEEKK/PEI blends decreased as the PEI content increased. The crystallinity of PEEKK was delayed by the presence of PEI and was attributed to an increased surface energy of the PEEKK crystals in the blends; the latter was supposed more defect-free than in pure PEEKK. However, the melting behavior of the PEEKK crystalline domains was unaffected by the blending, as the equilibrium T_m_ for the PEEKK in the blends was found to be close to the one for PEEKK alone, as measured by Zimmermann and Könnecke [108] and Wang et al. [116] on pure PEEKK.

## 7. Other PAEK Blends Obtained by Melt-Mixing

This section describes blends with other high-performance thermoplastics. Their miscibility depends on several factors, including the polymer’s structure, the mixing parameters or the blend composition. The whole literature reviewed is gathered in Table 1. PAEK/PAI and PAEK/PES give partially miscible blends in the melted state. The partial miscibility of PAEK/TPI and PAEK/LCP blends is discussed. PAEK/PBI and PAEK/PTFE blends are immiscible.

### 7.1. PAEK/PAI Blends

The miscibility of PEEK with PAI seems to depend on its molecular structure. Karcha and Porter [117] measured two distinct T_g_ at all compositions for PEEK/PAI blends, proving their immiscibility. Smyser and Brooks [118] patented, over 30 years ago, a method to compatibilize PEEK and PAI by adding various inorganic hydrates, such as zinc sulfate (ZnSO_4_·7H_2_O) or iron II sulfate (FeSO_4_·7H_2_O) and aluminum hydroxide (Al(OH)_3_) or cuprous hydroxide (CuOH). The hydrated blends may improve the injection molding process by preventing crosslinking between PEEK and PAI. They may also help to improve the mechanical properties to achieve better results than pure molded constituents. This method has been patented with other PAEK [119,120].

### 7.2. PAEK/PES Blends

Blends of PEEK and PES were reported to be either immiscible [121,122,123,124,125] or miscible [41,124,126], depending on how they were processed. Melt-mixed blends had two T_g_ for a processing temperature of 360 °C [121] or 350 °C [83,122,123] while Malik [126] measured single T_g_ for melt-mixed blends at 335 °C. This blend may undergo a low critical soluble temperature (LCST) at a temperature of around 340 °C [41,127]. Since the T_m_ of the PEEK is around 340 °C, the PEEK-rich phase begins to flow, hence having higher mobility, facilitating phase separation. Then, the high mobility of the PEEK overcomes the specific interaction between the sulfonate functions of the PES and the ether functions of the PEEK. Yu et al. [41] noted that postprocessing of the blends may alter their compatibility. Indeed, a single T_g_ was measured if the blends were processed using compression molding at 310 °C, while two T_g_, each close to that of the pure component, were measured for a postprocessing temperature of 350 °C.

It was reported in 30-year-old studies that an increase in the PES content reduced the crystallinity of PEEK/PES blends. Moreover, no crystallization of PEEK was observed above 70 wt.% of PES. For a wide range of compositions, no crystallization was noticed on quenched blends [121,124,126]. In recent work, Korycki et al. [125] showed the crystallinity in PEEK/PES blends above 70 wt.% of PEEK was unchanged, revealing that PES as a minor phase has no impact on the PEEK crystalline phase.

**Figure 17:**
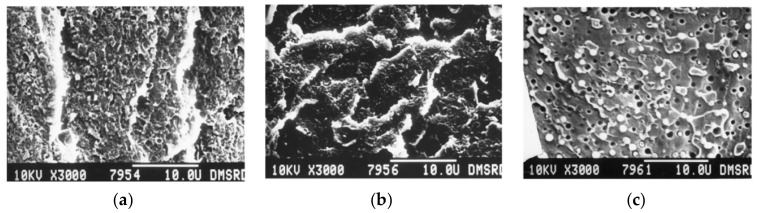
SEM fractography images of PEEK/PES blends obtained through melt-mixing then compression molding (**a**) 90/10 PEEK/PES (**b**) 75/25 PEEK/PES (**c**) 25/75 PEEK/PES, with permission from Wiley [127].

**Figure 18:**
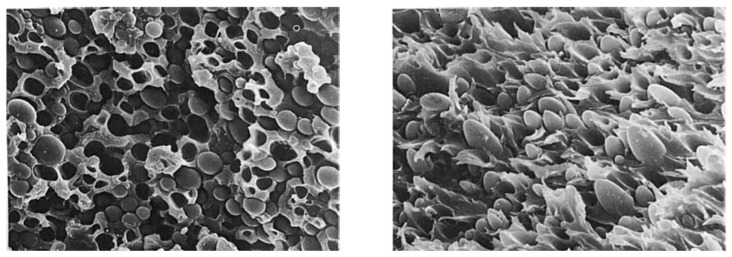
Morphologies of tensile fracture surface for 30/70 PEEK/PES compression-molded blends. Left: slowly cooled. Right: quenched, with permission from Wiley [128].

Malik [126] showed, using scanning electron microscopy (SEM), that PEEK forms well-dispersed spherical submicron domains in a PES matrix (20/80 PEEK/PES blend), which is the result of phase separation. However, the PEEK domains remained bonded during the fracture of films broken under nitrogen, supporting a good adhesion between phases. Nandan et al. [83,127] gave in-depth information about the morphology of those blends. For PEEK-rich blends (Figure 17a) before the phase inversion point, which is assumed to be at a 75/25 PEEK/PES composition, the PES is dispersed within the PEEK matrix in the form of submicron droplets. As the PES content increases (Figure 17c), the morphology changes to a two-phase structure, with PEEK droplets larger than a micron forming the dispersion. At the phase inversion point (Figure 17b), an almost co-continuous phase was observed. For cryogenically fractured blends at any composition, the dispersed phase was debonded from the matrix and deformed. The latter evidenced a good adhesion between the two phases. It shall be noted that Malik used a PEEK 380G, while all other studies used a PEEK 450G. Arzak et al. [128] noted a difference in the structure between slowly cooled and quenched blends. However, the structure of the quenched blend seems to account for a better phase adhesion than the slowly cooled blends, as fewer PEEK droplets debonded from the PES matrix are seen in the structure of the latter (Figure 18).

**Figure 19:**
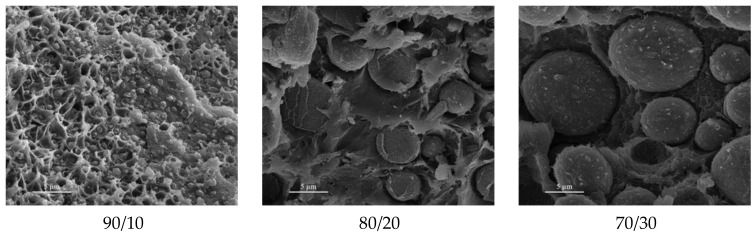
SEM images of the microstructure of PEEK/PES blends [wt.%] with phenolphthalein [125].

In order to compatibilize PEEK/PES blends, Korycki et al. [125] used phenolphthalein as a compatibilizer. The cardo side groups of phenolphthalein are supposed to be chemically bonded to PEEK and PES chains, increasing the interfacial adhesion between phases. The SEM analysis of fractured specimens displayed nano- to microsized PES spherical domains in a continuous PEEK phase, presented in Figure 19. The small pieces hooked on the surface of the PES droplets confirm the adhesion between both phases. It was noted that the crystallinity of PEEK was increased with PES content in the blends, demonstrating that despite phenolphthalein as a compatibilizer, the blends keep enough mobility to favor miscibility and PEEK crystallization. Among the two grades of PES tested with PEEK and phenolphthalein, the lowest molecular weight (PES 3010G) led to the highest crystallinity of PEEK, above 50%. Thus, the PEEK phase may be softened by the PES phase in the melted state, which gives macromolecules more mobility to self-organize into crystalline structures.

### 7.3. PAEK/TPI Blends

Blends of PEKK and TPI were defined as miscible at high and low TPI concentrations while being immiscible at intermediate (30 wt.% and 50 wt.%) compositions [30,129]. It was found that higher content of TPI favors miscibility, as the interaction parameter decreases with the TPI content increase. Moreover, the Fox equation was verified in miscible blends by the compositions and the T_α_ measured by DMTA [107,129]. Sauer et al. [18] provided a better understanding of the compatibility reasons for PAEK/TPI blends. By comparing PEEK, PEK and PEKK, it was shown that ketone linkages had an influence on miscibility with TPI. Indeed, it was hypothesized that the higher content of flexible ether linkages in PAEK, or their 120° angle, altered the ability to interact with the rigid polyimides. As a result, PEEK was immiscible with TPI but PEK and PEKK showed partial to full miscibility for high PAEK content. Looking further, a comparison of several PEKK with different terephthalic/isophthalic (T/I) content highlighted that a high fraction of 1.3 ketone linkage was thought to be more compatible with TPI, making PEKK (60T/40I) the most suitable for blending. Increasing the isophthalate moieties decreases the melting temperature and crystallization rate while increasing chain flexibility and retaining glass transition temperature at a high level. For PEKK content up to 20 wt.%, it acted as a high molecular diluent for strongly viscous polyimides [130]. More recently, Dominguez et al. [107] noted that semicrystalline blend miscibility undergoes the same phenomena as the mixtures of a semicrystalline polymer with an amorphous polymer. Thus, as PEEK crystallizes fast, the expelled TPI does not have enough time to reach the amorphous phase, creating phase separation. Also, PEKK crystallizes more slowly, giving more time for TPI to diffuse away from the crystal domains.

From a polarized optical microscope, the polyetheretherketone in PEEK/TPI blends seemed to crystallize as in neat PEEK, i.e., its crystalline structure is similar to that of neat PEEK. However, for high TPI content (above 60 wt.%), no PEEK crystallites were seen, indicating a hindering effect of the TPI on PEEK crystallization [131]. PEEK cold crystallization was also hindered by the TPI-rich phase [129]. Depending on the blend composition, both PEKK and TPI were able to crystallize during cooling from the melted state. Phase separation may also occur to some extent, as DMTA indicated the presence of two distinct amorphous phases in the melt crystallized blends, one rich in PEKK and the other rich in TPI.

To increase the compatibility between PEEK and TPI, Gao et al. [59] melt-mixed PEEK and TPI by adding synthesized optimized block length PEEK-*b*-TPI copolymers. The so-obtained compatibilized blends displayed a decrease in the interfacial tension and a narrowing of the T_g_ of both components. The impact of compatibilization on the crystallization of PEEK was not addressed, but since the segregation is reduced, it may hinder the PEEK crystallization.

### 7.4. PAEK/LCP Blends

Several studies have been conducted on melt-mixed PEEK/LCP blends. While Mehta and Isayev [132] obtained a blend behaving as pure PEEK, LCP seemed to promote PEEK hot crystallization, identically to a nucleating agent. Another work obtained nearly miscible-looking blends for more than 80 wt.% of PEEK but tending more toward a broadening of the T_g_ due to LCP crystal melting, which increased the amorphous population [133]. Partially or nonmiscible blends showed two distinct T_g_ affected by the other component, indicating the slight influence of LCP on PEEK [133,134].

Overall, the crystallinity of PAEK in immiscible blends was not affected by the presence of the other polymer for all PAEK/LCP blends reviewed. Thus, in immiscible blends, blended PAEK may crystallize as pure PAEK. However, Carvalho et al. [133] noted that in annealed PEEK/LCP, the melted LCP may act as an amorphous polymer and delay the PEEK crystallization.

### 7.5. PAEK/PBI Blends

**Figure 20:**
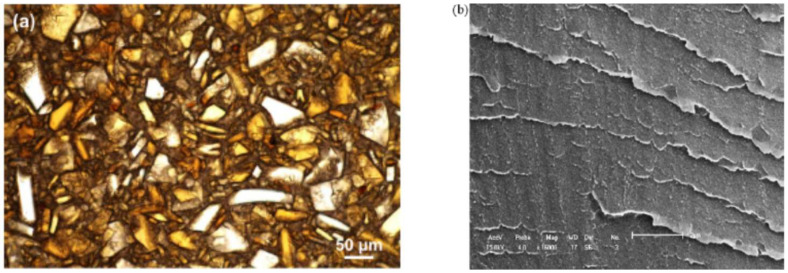
(**a**) Transmittance optical microscopy image of the melt-mixed PEEK/PBI blend with permission from ACS Publications [135]. (**b**) Scanning electron microscopy image of solution-blended SPEEK/PBI blend with permission from Elsevier [39].

Blends of PEEK and PBI have been shown to form immiscible blends [36,135]. PEEK can hardly be blended at temperatures above 420 °C because of its degradation, its T_m_ being around 340 °C. The glass transition temperature of PBI is 435 °C, so it remains solid during mixing. The obtained morphology is depicted in Figure 20a.

The presence of PBI affects the crystallization of the PEEK, as no spherulites are formed upon cooling [135]. While it was supposed that PEEK crystallization may be delayed by the PBI, no experiment supports this hypothesis, which is a question of interest that needs to be taken on.

### 7.6. PAEK/PTFE Blends

Some studies have reported the immiscibility of PEEK with PTFE [136,137]. Melt processable PTFE (MP-PTFE) is easier to process by melting thanks to the incorporation of perfluoropropylvinylether. Two distinct T_g_ were measured from its blend with PEEK (110 °C for the PTFE, 166 °C for the PEEK). SEM observation revealed a dispersed morphology of the minor phase in the rich phase and a bad adhesion between them. In the case of the PEEK-rich phase, MP-PTFE droplets had a size of over 100 microns. MP-PTFE copolymers irradiation using an electron beam created -COOH and -COF functional groups and enabled the compatibility of the blend components [137]. Indeed, the T_g_ of the PEEK phase was shifted toward a higher temperature (from 164 °C to 167 °C) for 50/50 compatibilized blends: the evidence of a better degree of miscibility. SEM images showed both a finer dispersion of PTFE and better adhesion between phases.

Stuart and Briscoe [6,136] showed that PTFE increased PEEK crystallinity. Using Raman spectroscopy, they found that the frequency of the carbonyl band shifted and that this band became narrower, hinting at PEEK macromolecules being more ordered. They later confirmed it [138] as a higher degree of order in the crystalline phase was discovered through X-ray diffraction.

## 8. Solution-Blending through Chemical Modification of Components

### 8.1. Solution-Blending with Sulfonated PEEK

Among the blends reviewed, solution blended SPEEK/PEI [139,140], SPEEK/PAI [117,140], SPEEK/PES [141] and SPEEK/PBI [39,142,143] were reported to be miscible at all compositions. For all the blends, a single T_g_ with a positive deviation from the additivity law was measured, suggesting the presence of specific interactions.

**Figure 21:**
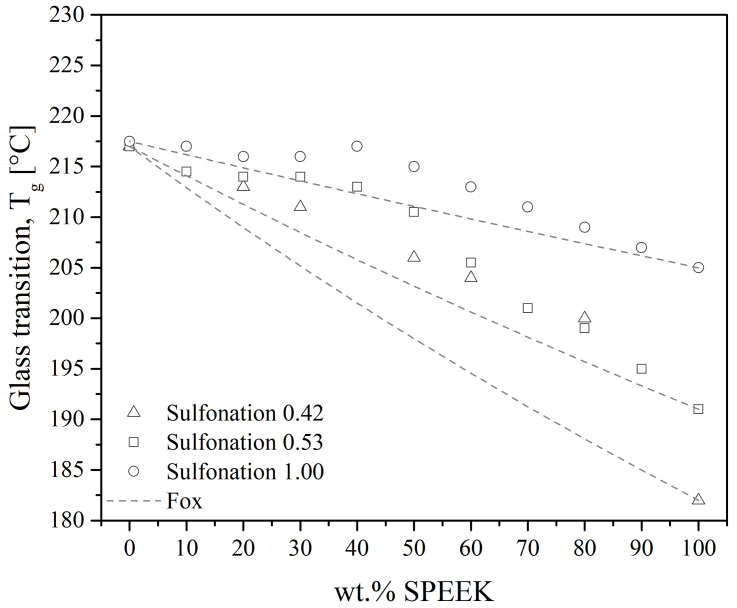
Glass transition temperature as a function of SPEEK content in SPEEK/PEI blends. Points represent experimental data from the literature [117,140], solid lines represent the Fox equation’s trend.

Karcha and Porter [117,140] measured a single, sharp T_g_ at all compositions and sulfonation levels, presented in Figure 21. However, the T_g_ has deviated from the Fox equation. They suggested that the sulfonate functions of the sulfonated PEEK added strong intermolecular interactions. The same authors also studied SPEEK/PAI blends, which were found to be also miscible at all compositions. Using a Fourier transform infrared (FTIR) spectrometer, they identified that the specific interaction is the formation of intermolecular electron donor-acceptor complexes between the substituted phenylene rings of the SPEEK and the N-phenylene units of the PAI. The sulfonated group of the SPEEK acts as the electron acceptor, which helps in the formation of the complexes. Indeed, PEEK and PAI are not miscible in the melt state. Thus, the formation of such complexes by sulfonation of the PEEK decreases the free energy of mixing to the point of giving a miscible SPEEK/PAI blend. In the case of SPEEK/PBI blends, the underlying specific interaction is reported to be an acid-base interaction. Specifically, sulfonated groups of the SPEEK are acidic. They can form proton exchange complexes with the amino units of the PBI, the latter with the role of proton donor. Since SPEEK/PBI blends are mostly prepared for use in exchange membranes, most research focuses on their improved thermal and chemical stability [39,99,142,144,145,146]. These features may be explained by the morphology of the blends. As shown in Figure 20b, the PBI is well dispersed in the SPEEK matrix. Figure 20a shows the morphology of a melt-mixed PEEK/PBI blend, where immiscibility appears through different separated phases. Thus, good dispersion arises from the acid-base complexes formation.

### 8.2. Crystallization of SPEEK Blends

Reports about SPEEK/PEI blends have not assessed the crystallinity of the blends. Nevertheless, nanoscale transparency has been reported for SPEEK/PAI blends [33,141]. DSC traces of SPEEK/PBI [142] show no melting peak, hinting at a reduction in SPEEK crystallization. Wu et al. [33,141] supposed that SPEEK crystallinity would be greatly reduced when blended with PES while investigating the water and solvent uptake of SPEEK/PES blends.

Sulfonation has a twofold effect in SPEEK-based blends: (i) on the one hand, it reduces the intrinsic PEEK crystallinity [94], thus decreasing intermolecular (i.e., PEEK-PEEK) interactions; (ii) on the other hand, it increases the specific interactions through the formation of electron/proton donor-acceptor complexes. Both cases point toward the same direction, which is a reduction in crystallinity.

Overall, in-depth investigations would be required to make the distinction between sulfonation and the presence of an amorphous HPT on SPEEK crystallinity (such as blending para-PEI with SPEEK), to elucidate the structure–properties relationship behind the absence of crystallinity in miscible SPEEK/HPT blends, in order to enlighten and add to a deeper understanding of the nature of miscible blends.

## 9. Effect of Blends on PAEK’s Mechanical Properties

### 9.1. Blends Obtained by Melt-Mixing

Mechanical properties of blends display various results depending on their thermo-mechanical history, whether they have been cooled rapidly to get amorphous blends or crystallized through annealing. Additivity rules are expected to fit well the experimental data for miscible blends, such as the properties that would follow a mixing law. Deviations from additivity may indicate that the phases in the blends, either:

- do not adhere to each other, leading to a negative deviation;
- or, strongly adhere, yielding a positive deviation, mainly attributed to specific interactions in the blend.

Semicrystalline PAEKs are more brittle than amorphous PAEKs due to the higher stiffness of the crystalline network compared to amorphous ones [27]. This section reports the results of the mechanical characterization of PAEK/HPT blends. However, among all the articles reviewed, a wider number of studies deal with PEEK/PEI blends. The blend preparation for some examples is presented in Table 2. It is known that the processing temperature may affect the miscibility properties of blends. The mechanical properties of the other types of blends are mentioned at the end of this section.

**Figure 22:**
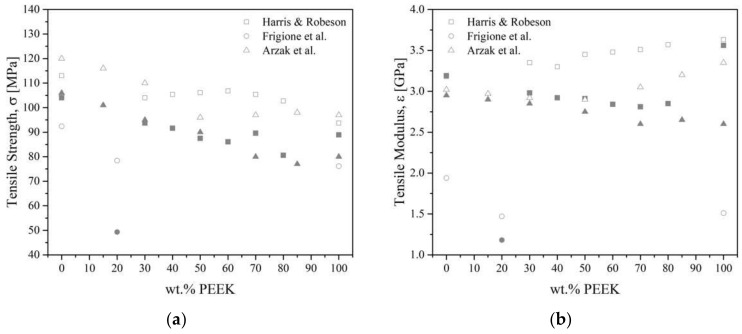
The tensile strength (**a**) and modulus (**b**) as a function of the volume fraction of PEEK for PEEK/PEI blends from the literature [27,111,147]. Empty symbols for annealed blends, full symbols for as-molded blends.

The general trend for PEEK/PEI blends follows that of PEEK, i.e., annealed blends are more brittle than amorphous ones. Harris and Robeson [27] noted that the toughness of PEEK/PEI blends passed through a maximum of 60 wt.% of PEEK when measured by tensile impact strength. As-molded pure PEEK had an estimated toughness of 279 kJ.m^−2^, whereas blends with 60 wt.% of PEEK had more than a 60% increase with a 414 kJ·m^−2^ toughness. Annealed blends followed the same trend, with a toughness of 254 kJ·m^−2^ for blends with 80 wt.% of PEEK compared to 170 kJ·m^−2^ for neat PEEK. Moreover, tensile strength goes through a maximum of 106.8 MPa for 60 wt.% of PEEK for an annealed blend. As-molded (i.e., crystallinity is not controlled) shows negative deviation and annealed shows positive deviation (probably causes crystalline network reinforcement, annealing relieves stress according to the authors) [27,111,147]. Goodwin et al. [100] also noted some deviation from linearity when measuring the flexural modulus as obtained from DMTA for PEEK/PEI blends. The 40 wt.% of PEEK had a modulus of 2.8 GPa, while pure PEEK had 4 GPa of modulus. Going through a minimum of 80 wt.% of PEEK indicates specific interactions may take place at the interface, not explained by the authors. The mechanical properties of PEEK/PEI blends are complicated by the crystallinity of molded samples containing greater than 70 wt.% of PEEK [27]. Above 70 wt.% of PEEK, the authors highlight the brittleness of the blends. Harris et al. [151] noted such deviation for both PEEK/PEI (minimum at 40 wt.% of PEEK) and PEK/PEI (minimum at 60 wt.% of PEK) blends. Frigione et al. [111] reported that crystallized PEEK/PEI 20/80 blends had similar or slightly weaker mechanical properties to the tensile strength of 78.4 MPa and modulus of 1.47 GPa compared to pure PEEK, while the as-molded blend was much lower with 19.3 MPa and 1.18 GPa, respectively, Figure 22.

Additionally, after annealing, blends have better mechanical properties (Young’s modulus and tensile strength) than as-molded blends, as noted by Arzak et al. [147]. Their tensile modulus and tensile strength deviated from the linearity predicted by the mixing rule because of crystallinity development. Though crystallinity gives an overall increase in rigidity, the incorporation of PEI diminished the overall quantity of PEEK crystals, hence a negative deviation from additivity. Also, the ductility of the blends has a more complex behavior. Pure annealed PEI and PEEK have a ductility of 10% and 25%, respectively. The maximum 55% is observed at 50/50 PEEK/PEI composition in annealed blends, while the ductility of as-molded blends appears fairly uniform and slightly enhanced concerning the additive rule of mixtures. PEI increases the ductility of the blends from 45% to 55%, balancing the loss of properties. This balance of opposite trends was of greater importance at temperatures close to the T_g_ of the blends. Annealed blends tested at 125 °C exhibited strong deviations, while as-molded blends showed no variation or followed additivity. However, the ductility was more pronounced in annealed blends, thus rendering them usable at this range of temperature. Thus, blending with PEI has the additional effect that some crystalline PEEK is converted to the more ductile and less stiff amorphous PEEK, which gives rise to higher ductilities and a smaller modulus of elasticity and tensile strength in the blends than the pure materials. Gensler et al. [152] noted for thin PEEK/PEI films a brittle-ductile transition with increasing temperature for blends containing up to 60 wt.% of PEEK. However, blends containing more PEEK showed no transition, as PEEK crystallization was strain-induced, preventing disentanglement and further crazing. The 50/50 PEEK/PEI blends were also reported to have reduced solvent (acetone) uptake compared to pure components [153]. The acetone uptake induces a phase swelling, resulting in the recrystallization of PEEK. The mechanical properties (tensile strength, yield stress, Young’s modulus and strain at break) of the blends were also affected by the presence of the solvent. Overall, Young’s modulus was more sensitive to plasticization and dropped from 1340 MPa to 500 MPa, while the tensile strength (decrease from 70 MPa to 16 MPa) and the strain at failure (decrease from 50% to 17%) were more sensitive to the degree of crystallinity.

To sum up, PAEK and PEI are miscible; all the authors presented better tensile strength and Young’s modulus for annealed blends than for as-molded ones. The balance of properties changed in annealed blends because of the change in crystallinity with composition, giving clear trend changes at the intermediate composition, where crystallinity content also clearly changed.

**Figure 23:**
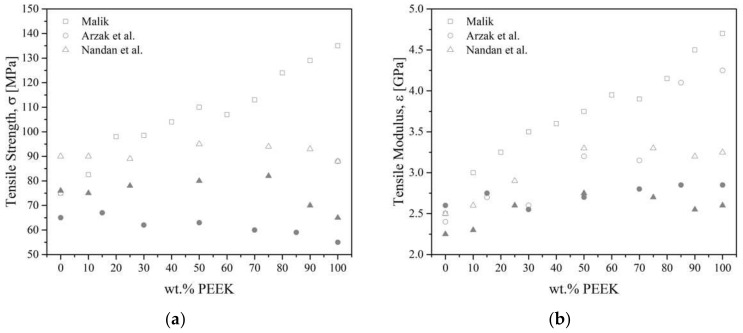
The tensile strength (**a**) and modulus (**b**) as a function of the volume fraction of PEEK for PEEK/PES blends from the literature [121,123,126]. Empty symbols for annealed or slowly cooled blends, full symbols for quenched blends.

As a reminder, PEEK is not miscible with PES. Young’s modulus of PEEK/PES blends increases with increasing PEEK content [121,123,126,128]. All authors agreed on the fact that blends containing more than 60 wt.% of PEEK followed a mixing rule, despite using a different grade of PEEK (450G and 380G, respectively, by Azrak et al. [121,128] and Malik [126], while Nandan et al. [83,122,123,127] synthesized their own PEEK). Quenched [121] blends followed a mixing rule, while annealed [128] or slowly cooled blends [121] exhibited a negative deviation, Figure 23. The deviation was the result of the development of crystallinity. Malik [126] and Nandan et al. [123] measured a positive deviation from linearity, with a maximal modulus at 40–50 wt.% of PEEK content. Good adhesion and synergistic interaction, such as the increased density of chain entanglement [16,123] between the PEEK and the PES were thought to be responsible for this positive deviation. Azrak et al. [121] measured the yield stress for the blends that followed a linear behavior with the concentration in the case of quenched blends. Slowly cooled blends were brittle; for example, they broke before reaching the yield point. The crystallized PEEK increases the brittleness of the blends. Nandan et al. [123] measured elongation at break that deviated negatively for all compositions except for 90/10 PEEK/PES, which showed a positive deviation due to an important synergistic effect at this composition with ductile behavior.

PEEK and PES are not miscible from a thermodynamic point of view. However, some compatibility may be achieved for particular blending conditions and cooling phases. Compatibility arises from specific interaction between the PES sulfonated function and the PEEK backbone, hence the mechanical properties slightly deviating from additivity. Annealed or slowly cooled blends result in a higher tensile strength and Young’s modulus than quenched blends.

Next, blends of TLCP with PEEK reported by Kiss [48] show that PEEK modulus increased from 3.5 GPa to 4.3 GPa with the addition of 30 wt.% of TLCP; however, the tensile strength and strain to failure decreased from 84.1 MPa to 71.7 Mpa and from 48% to 2.5%. Mehta and Isayev [132] noted that LCP may enhance PEEK’s mechanical properties. The flexural modulus as obtained from DMTA, increased by an increasing LCP concentration, indicating the reinforcement of PEEK with LCP. Miao et al. [148] reported an increase in the tensile modulus and tensile strength from 1.85 GPa to 2.42 GPa and from 80.8 MPa to 110 MPa with only 2 wt.% of LCP.

Liu et al. [135] measured Young’s modulus of 5.4 GPa for PEEK/PBI blends, which was close to the value reported by Alvarez and DiSano [149] of 5.1 GPa. In their work, they produced PEEK/PBI blends through extrusion and compression molding. PBI and PEEK are known to be incompatible with each other in the dry state. The possible influence of hygrothermal exposure on interfacial bonding between PEEK and PBI has been studied. The blends of PEEK/PEI 50/50 were treated under different conditions. Dry and water-saturated at 60 °C blends demonstrate an increase in the tensile strength from 122 MPa to 127 MPa, while the blend treated in hot water at 288 °C reached only 56 MPa. Alvarez and DiSano [149] reported that both compressive and tensile properties were improved by the incorporation of PBI into the PAEK. The tensile strength of pure PEK and PEEK were 102 MPa and 97 MPa while their blends with 50 wt.% of PBI reached 119 MPa and 125 MPa, respectively.

PTFE is usually incorporated into PEEK to target tribological applications. Indeed, PTFE implied a reduction in the coefficient of friction compared to pure PEEK [1,150,154] and it implied a loss of wear. The PTFE is generally thought to migrate to the surface of the blend [6,155]. Thus, if the blends contain too much PTFE, its surface consists only of PTFE. An optimal PTFE concentration has been found between 10 wt.% and 20 wt.%, depending on the molecular weight of the PEEK used [136,154,155] and the process used to elaborate the blends. Plasma treatment before blending may improve the tribological properties, as plasma-treated blends had a decreased specific wear rate and sliding friction coefficient [156]. Blends of irradiated MP-PTFE showed better mechanical properties than PEEK/PTFE blends. Indeed, a tripled strain at break and a doubled stress at break were reported for the former blend [137]. Bijwe et al. [150] noted that the incorporation of PTFE may deteriorate the mechanical properties of PEEK, rendering the PEEK/PTFE blend relevant for only a few tribological applications. So, the tensile strength for pure PEEK of 87 MPa decreased to 64.7 MPa for a blend with 30 wt.% of PTFE, while the tensile modulus changed from 3.9 GPa to 1.23 GPa, respectively.

Thus, TLCP and PBI, despite the lack of miscibility with PAEK, can be compatible in some blend compositions, achieving a higher tensile strength and modulus as blends than pure PAEK. The special case of PAEK/PTFE blends attracted our attention, in terms of that those polymers are highly immiscible but their blends give the foreseen properties, especially a reduced friction coefficient. Indeed, the incorporated PTFE migrates to the PEEK surface acting as a lubricant. PTFE was also found to increase PEEK crystallinity but an in-depth reason was not given to explain the observed trend.

### 9.2. Performances of Blends Obtained by Solution Blending

SPEEK is mainly blended with HPT to elaborate exchange membranes. A proton exchange membrane fuel cell transforms the energy liberated during the hydrogen and oxygen reaction from chemical to electrical energy. The sulfonation of PEEK allows its proton conductivity to be increased [35,40]. Electrochemical properties (i.e., water uptake, thermal stability, proton exchange, dielectric conductivity) are the most reported properties. Most SPEEK/HPT blends aim to create a cost-effective solution as an alternative membrane to Nafion^®^. The mechanical properties of such membranes have barely been studied.

Daud et al. [157] reported that SPEEK/PES had performances comparable to commercial ones. The presence of the ionic sulfonate groups increased the hydrophilic nature of the blends and increased the surface charges [158]. The mechanical properties reported in such studies cannot be compared due to varying water uptake. According to Arigonda et al. [96], SPEEK/PES demonstrates a lower Young’s modulus for dry blends of 3.6 MPa and 2.2 MPa for 20 wt.% and 40 wt.% of PES, respectively, than pure SPEEK of 4.8 MPa. Hydrated SPEEK had a three-fold decrease in its modulus of 1.5 MPa, whereas the SPEEK/PES blends retained their mechanical properties of 2.6 MPa and 2.4 MPa for 20 wt.% and 40 wt.% of PES, respectively.

## 10. Ternary and Compatibilized Blends

Ternary blends may be relevant as the third component and may act as a link between two incompatible materials, provided it is miscible with both.

The properties of PAEK arise mainly from a difference in the molecular arrangement of the phenyl unit, which in turn influences their melting point and crystalline structure. The advantages of blending PAEK altogether reside in the fact that PAEK blends are isomorphic. They exhibit a single T_g_ and T_m_. The high ethers-to-ketones ratio of PAEK has limited use in injection molding, as their T_m_ is too close to their degradation temperature. Blending those PAEK with lower T_m_ PAEK enables them to be processed at a lower temperature while keeping their outstanding properties.

Harris et al. [159] blended various PAEK (such as PEK/PEKK) and found a single T_g_, a single T_m_ and mechanical compatibility, indicating isomorphism. They also prepared blends with random copolymers composed of different PAEK, such as a PEKK-*g*-PEEK copolymer, which was also isomorphic when blended with other PAEK. Some blends were not isomorphic when in a binary mixture but were isomorphic when a third PAEK was added, the latter forming binary isomorphic blends with the two other components. Thus, the PEKK-*g*-PEEK copolymer acted as a compatibilizer. Still keeping with the same idea, Gao et al. [59] observed an increased flexural modulus (from 3280 MPa to 3314 MPa) and elongation at break (from 8.6% to 27.4%) for the compatibilized blends compared to uncompatibilized ones. For an optimized block length copolymer, they recorded a 200% improvement in terms of elongation at break. Dominguez et al. [107] showed that an increase in the crystallinity in such ternary blends induced an increase in T_g_ but a decrease in rubbery moduli.

Dawkins et al. [160] prepared PBI/PAEK/PEI blends through both solution-blending and mix-melting. They measured only two T_g_ as the PAEK would be miscible with the PEI but not with the PBI. The second T_g_ was always close to the T_g_ of the PBI. Interestingly, both methods yielded a first T_g_ in agreement with that of binary PEEK/PEI blends, highlighting incompatibility between the PEEK and the PBI and a possible incompatibility of PEI with PBI.

Chen et al. [161] prepared miscible PEEK/PEI/PPS blends and they reported a homogeneous morphology [104]. The unique T_g_ of the blends was higher than that of pure PEEK. The crystallinity of PEEK was also higher than that of pure PEEK, with a maximum crystallinity of 37.8% for 60/30/10 PEEK/PEI/PES blends. Regarding the mechanical properties, they focused on tribology: higher coefficients of friction and wear rates were recorded for the blends compared to pure PEEK.

PAI/SPEEK/PEI blends were investigated by Karcha and Porter [162] since they studied the miscible binary blends SPEEK/PAI and SPEEK/PEI. The immiscibility of PEI and PAI makes SPEEK a suitable candidate as a compatibilizer. Only a few compositions lead to miscible blends both before and after annealing. The composition with up to 10 wt.% of PAI was miscible for the degree of sulfonation of 0.53, whereas, with the degree of sulfonation of 1.00, the blends containing up to 20 wt.% of PAI and PEI were miscible (i.e., 20/40/20 PAI/SPEEK/PEI).

**Figure 24:**
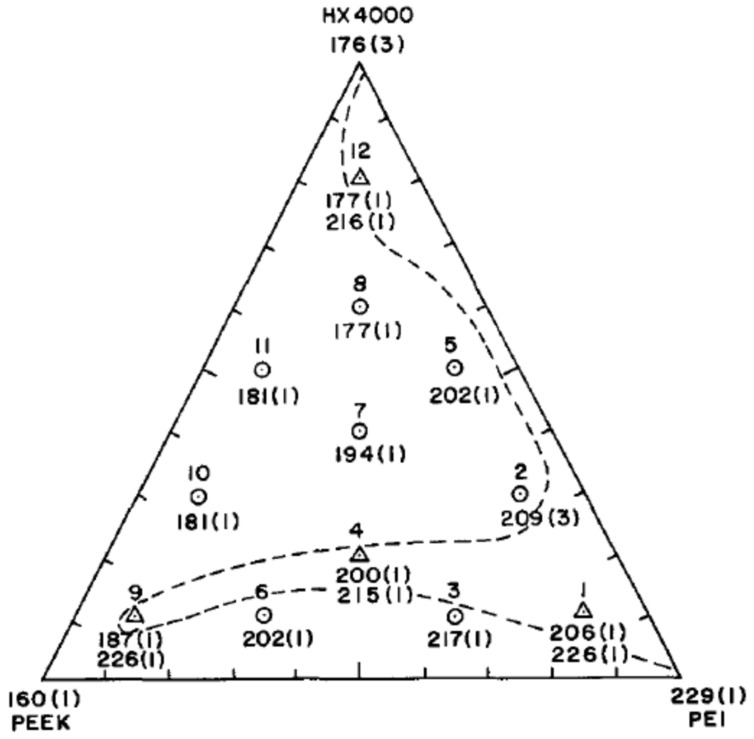
Phase diagram of T_g_ obtained by DSC of PEEK/PEI/LCP(HX400) blends after annealing, where ○ is composition with one T_g_, Δ is composition with two T_g_, --- is approximate boundary between composition with one and two T_g_, standard deviations are given in brackets, with permission from Elsevier [163].

Based on the consideration that PEI/LCP blends exhibit compatibility and synergistic effects and that PEEK and PEI are miscible, Bretas and Baird [163] explored the possibility of blending LCP with PEEK with PEI as compatibilizer. The ternary PEEK/PEI/LCP blend exhibited compatibility both before and after annealing. In the latter case, the blends were more stable and some compositions showed miscibility. Either blend with a high content of LCP (such as 10/10/80 PEEK/PEI/LCP blends) or with a high content of PEI (such as 10/80/10 PEEK/PEI/LCP blends) showed compatibility. Figure 24 shows the complexity of these blends, where it is easy to see that miscibility is not related to composition in a straightforward way, even though blends with low LCP content tend to be miscible [164] as they were mostly composed of PEEK and PEI. Specific interactions such as dipole–dipole interaction between some carbonyl groups were given causes for the observed miscibility. The link between the mechanical properties and composition was complex but overall high LCP content blends had a higher modulus and high PEI and PEEK contents were more ductile (i.e., high tensile strength and high strain at failure), with more PEI leading to higher values.

## 11. Conclusions

Like conventional polymer blends, those of high-performance polymers are seldom naturally miscible. Among the blends reviewed, PEEK/PEI is the only one that gives miscible blends when the blends are simply elaborated through melt-mixing. Other PAEK are partially miscible with PEI; their degree of miscibility depends on the composition. The degree of miscibility of PAEK/PAI and PAEK/TPI depends on the ketone linkages of the PAEK, while PAEK/PBI and PAEK/PTFE are immiscible. PAEK/PES blends are miscible if the processing temperature is below a given threshold.

Another way of preparation is solution blending, meaning dissolving both polymers in a suitable solvent. Those PAEK/HPT blends with the HPT aforementioned enable a higher degree of miscibility. In particular, solution blending of sulfonated PAEK enabled high miscibility with PEI, PAI, PBI, TPI and PES thanks to the specific interactions between the sulfonate groups of the PAEK and mainly the imide functions (respectively, the sulfonate group of the PES).

The mechanical properties of the blends are not systematically reported in the literature reviewed, but instead, the properties relevant to the specific end-use envisioned are. The mechanical properties were reported for PAEK/PEI and PAEK/PES blends, as the primary function of the amorphous polymers was to enhance PAEK processability and to enable its use above its T_g_. Thus, the mechanical properties of those blends were of utmost importance. Reports found that in the amorphous state, the overall properties decreased concerning those of PAEK, while semicrystalline blends had properties similar to that of neat PAEK. Those properties are not reported in the case of PAEK/PAI, PAEK/PBI and PAEK/TPI. The primary role of the PAEK in such studies is to reduce water uptake and increase proton conductivity. In this scope, sulfonation of PAEK was required, and miscible SPAEK/PBI and SPAEK/TPI are mentioned as serious candidates for PEMFC membranes.

Similarly, few reports have focused on the morphology of the obtained blends, mainly because their aimed properties were achieved, especially for the blends used in membranes.

Compatibilization of polymer blends is widely used for low-performance thermoplastic polymers, such as anhydride maleic acid being used to compatibilize PET/PP blends. This route remains to be explored for HPT blends, and the importance of doing so will inevitably increase. Indeed, most of the partial blends overviewed are obtained through solution blending, using highly concentrated strong acids, producing large quantities of waste with a high environmental impact. Indeed, solution blending does not fall under the latest environmental standards such as the REACH regulation, which aims to improve the protection of human health and the environment from the risks of chemicals. Producing compatible HPT blends respecting those standards will be one of the next challenges to overcome shortly. The first answer would be the study of ternary- or multicomponent blends. The most interest in the multiphasic system would be the fine control of the morphology, especially targeting the selective location of the components. This kind of system has already been extensively studied for low-performance thermoplastic alloys but seldom reported for HPT blends. Control of the morphology may also be realized by controlling the processing; it has been demonstrated that some immiscible blends, such as PEEK/PES, may be mechanically compatible. This path deserves a thorough and systematic investigation, the control of morphology being little studied in the literature reviewed.

The future trends will deal with the characterization of the spatial distribution of each phase in PAEK-based blends. More progress is necessary to give a fine mapping of the spatial distribution of the morphology at the surface and inside the volume of blends. The crystallization of polymer chains confined in nanodomains has not been resolved for PAEK blends until now. Also, the synthesis of tailored copolymers will be useful to improve the miscibility of PAEK-based polymer blends. Another future challenge will be the manufacturing of composites with PAEK-based blends as matrices in order to benefit from the remarkable properties of these blends.

Interest in HPT blends continues to grow as they represent an alternative to metals to face the upcoming environmental and energy transition. This interest will only grow when the above challenges are met.

## Figures and Tables

**Table 1:** Classification of the most common HPT blended with PAEK as a function of their miscibility and method of blending.

Blending Method	Miscible Blends	Partially Miscible Blends	Immiscible Blends
Melt-mixing	PAEK/*meta*-PEI	PAEK/PAI	PEEK/*para*-PEI
		PAEK/PES	PEEK/*ortho*-PEI
		PEKK/TPI PEK/TPI	PEEK/TPI
		PAEK/LCP	PEEK/PBI
			PAEK/PTFE
Solution blending	SPEEK/PAI	PEKK/PBI	
	SPEEK/PEI	PEEK/PES	
	SPEEK/PBI		
	SPEEK/PES		

**Table 2:** Summary of the PAEK-based blend preparation with different additives.

Grade of PAEK	Tensile Strength [MPa]	Grade of HPT	Tensile Strength [MPa]	Blend and Sample Preparation	Processing Parameters	Max Tensile Strength of Blends [MPa]	Ref.
PEKK (synthesized)	93.7	PEI Ultem 1000	113	Extrusion (Ex) and injection molding (IM) or compression molding (CM) Annealed	360–380 °C 370–380 °C 370–390 °C 200 °C/2 h	106.8 (60/40)	[27]
PEEK 450G	76.1	PEI Ultem 1000	92.4	Mixing (Mx) Annealed	365 °C/5′ 300 °C	78.4 (20/80)	[111]
PEEK 450G	97	PEI Ultem 1000	120	Dry Mx IM Annealed	370 °C 185 °C/24 h	116 (15/85)	[147]
PEEK 450G	135	PES 4100	72	Mx CM	355 °C/10′ 355 °C	129 (90/10)	[126]
PEEK 380G	72.5	PES Ultrason E-2000	89	Mx IM	360 °C/12′ 370 °C	/	[121]
PEKK (synthesized)	89	PES Radel A-300	90	Mx Annealed	350 °C/30″ 185 °C/24 h	95 (50/50)	[123]
PEEK	84.1	LCP polyester	235.1	Ex IM	/ /	71.1 (70/30)	[48]
PEEK	80.8	LCP coPAEK (synthesized)	/	Ex IM	350 °C 350 °C	110 (98/2)	[148]
PEEK	/	PBI	/	Ex CM	385–425 °C 420 °C/30′	127 (50/50)	[135]
PEEK	97	PBI	/	Ex IM	455–510 °C 385–455 °C	125 (50/50)	[149]
PEEK Gatone 5400	87	PTFE	/	Ex IM	330–350 °C /	84 (92.5/7.5)	[150]

## Data Availability

Not applicable.

## Nomenclature of Polymers

PAEK	Polyaryletherketone	HPT	High-performance thermoplastic
PEKK	Polyetherketoneketone	PEI	Polyetherimide
PEKEKK	Polyetherketonetherketoneketone	PAI	Polyamideimide
PEK	Polyetherketone	PBI	Polybenzimidazole
PEEKK	Polyetheretherketoneketone	PES	Polyethersulfone
PEEKEK	Polyetheretherketonetherketone	TPI	Thermoplastic polyimide
PEEK	Polyetheretherketone	PTFE	Polyterafluoroethylene
PEEEK	Polyetheretheretherketone	LCP	Liquid crystal polymer
SPEEK	Sulfonated polyetheretherketone	TLCP	Thermoplastic liquid crystal polymer
